# Concurrent affective and linguistic prosody with the same emotional valence elicits a late positive ERP response

**DOI:** 10.1111/ejn.14658

**Published:** 2020-01-29

**Authors:** Hatice Zora, Mary Rudner, Anna K. Montell Magnusson

**Affiliations:** ^1^ Department of Linguistics Stockholm University Stockholm Sweden; ^2^ Department of Behavioral Sciences and Learning Linköping University Linköping Sweden; ^3^ Department of Clinical Science, Intervention, and Technology Karolinska Institutet Stockholm Sweden; ^4^ Department of Biomedical and Clinical Sciences Linköping University Linköping Sweden

**Keywords:** electroencephalography, emotion, event‐related potential, language, prosody

## Abstract

Change in linguistic prosody generates a mismatch negativity response (MMN), indicating neural representation of linguistic prosody, while change in affective prosody generates a positive response (P3a), reflecting its motivational salience. However, the neural response to concurrent affective and linguistic prosody is unknown. The present paper investigates the integration of these two prosodic features in the brain by examining the neural response to separate and concurrent processing by electroencephalography (EEG). A spoken pair of Swedish words—[ˈfɑ́ːsɛn] phase and [ˈfɑ̀ːsɛn] damn—that differed in emotional semantics due to linguistic prosody was presented to 16 subjects in an angry and neutral affective prosody using a passive auditory oddball paradigm. Acoustically matched pseudowords—[ˈvɑ́ːsɛm] and [ˈvɑ̀ːsɛm]—were used as controls. Following the constructionist concept of emotions, accentuating the conceptualization of emotions based on language, it was hypothesized that concurrent affective and linguistic prosody with the same valence—angry [ˈfɑ̀ːsɛn] damn—would elicit a unique late EEG signature, reflecting the temporal integration of affective voice with emotional semantics of prosodic origin. In accordance, linguistic prosody elicited an MMN at 300–350 ms, and affective prosody evoked a P3a at 350–400 ms, irrespective of semantics. Beyond these responses, concurrent affective and linguistic prosody evoked a late positive component (LPC) at 820–870 ms in frontal areas, indicating the conceptualization of affective prosody based on linguistic prosody. This study provides evidence that the brain does not only distinguish between these two functions of prosody but also integrates them based on language and experience.

## Graphical abstract

Prosody—a natural component of all languages—conveys both higher‐order linguistic and lower‐order affective voice cues. Here, the neural response to separate and concurrent linguistic and affective prosody with the same valance (angry) was investigated by electroencephalography (EEG). A late frontal EEG response emerged only with concurrent linguistic and affective prosody, indicating that lower‐ and higher‐order brain activity is integrated for the emotional conceptualization of language.

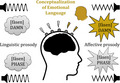

**Abbreviations**
CSM: Common mode sense
DRL: Driven right leg
EEG: Electroencephalography
ERP: Event‐related potential
f0: Fundamental frequency
fMRI: Functional magnetic resonance imaging
ICA: Independent component analysis
ISI: Interstimulus interval
LPC: Late positive component
MMN: Mismatch negativity
P3a: Positive event‐related potential response
ROI: Region of interest
SOA: Stimulus onset asynchrony
SPL: Sound pressure level

## 1 INTRODUCTION

Prosody—variations in physical properties of the auditory signal such as duration, intensity, and fundamental frequency (*f*0)—is crucial for spoken communication and conveys linguistic functions such as semantics and syntax (for a review, see Wagner & Watson, 2010). Linguistic prosody has previously been investigated using the mismatch negativity (MMN) component of event‐related potentials (ERPs), which signals the brain's automatic reaction to any change in the auditory sensory input, and is elicited irrespective of the subject's attention and behavioral discrimination (Alho & Sinervo, 1997; Näätänen, Gaillard, & Mantysalo, 1978; Näätänen, Paavilainen, Rinne, & Alho, 2007; Näätänen & Winkler, 1999; Paavilainen, Arajarvi, & Takegata, 2007; Pulvermüller & Shtyrov, 2006). The fronto‐centrally maximal MMN component, with a typical latency range of 150–250 ms after change onset, has been an objective and useful tool for investigating both low‐level sensory and high‐level cognitive prosodic processing (Friederici, Friedrich, & Christophe, 2007; Näätänen et al., 1978, 2007; Näätänen & Winkler, 1999; Weber, Hahne, Friedrich, & Friederici, 2004; Zora, Heldner, & Schwarz, 2016; Zora, Riad, & Ylinen, 2019; Zora, Schwarz, & Heldner, 2015). Zora et al. (2015), for instance, investigated the neural correlates of linguistic prosody and its contribution to semantic processing in the English stress‐contrastive verb–noun pair, upˈset versus. ˈupset, along with the pseudoword control, ukˈfet versus. ˈukfet. The results indicated larger MMN response to a linguistic prosody change in words than in pseudowords, reflecting not only sensory prosodic processing but also cognitive prosodic processing due to the association between linguistic prosody and semantics.

The MMN response is typically followed by a fronto‐centrally maximal P3a response, reflecting an involuntary attention switch to the auditory change, which indexes salience and contextual novelty of stimuli (Escera, Alho, Winkler, & Näätänen, 1998; Escera & Corral, 2007; Näätänen et al., 2007; Polich, 2007). The P3a response has especially been shown to be sensitive to prosodic salience (Wambacq & Jerger, 2004; Wang, Friedman, Ritter, & Bersick, 2005). Prosodic changes in unattended speech sounds have, for instance, been shown to capture more attention compared to temporal changes, and, therefore, elicit a P3a response (Wang et al., 2005). Similarly, a larger P3a response has also been shown with affective prosody compared to with neutral prosody (Carminati, Fiori‐Duharcourt, & Isel, 2018; Pakarinen et al., 2014), probably not only due to the acoustic salience but also due to the motivational salience of affective prosody (Wambacq & Jerger, 2004; Wang et al., 2005; see also Bradley, Codispoti, Cuthbert, & Lang, 2001; Bradley et al., 2003; Schupp et al., 2004). The affective function of prosody differentiates well among emotional expressions and is equally important for spoken communication. Previous ERP research has provided evidence for the integration of affective prosody and emotional semantics for an efficient communication of auditory emotional sentences (Kotz & Paulmann, 2007; Paulmann & Kotz, 2008; Wambacq & Jerger, 2004). Concurrent affective prosody and emotional semantics with the same valence have, for instance, been shown to elicit a larger P3a response than either affective prosody or emotional semantics alone, reflecting the automatic integration of emotional information (Wambacq & Jerger, 2004).

A major issue in comprehending speech communication is to determine how the brain handles multiple prosodic cues concurrently to extract information from speech that is encoded linguistically and affectively. In the present paper, we examined the integration of these prosodic cues by investigating the pre‐attentive neural response to concurrent and separate processing of, respectively, affective and linguistic prosody. To this end, a Swedish word pair where the linguistic prosody modulates the emotional semantics—‘fasen’ [ˈfɑ́ːsɛn] *phase* and ‘fasen’ [ˈfɑ̀ːsɛn] *damn*—were investigated once with an angry and once with a neutral affective prosody. Anger was chosen since the recognition of vocal anger tends to be higher than that of other emotions irrespective of language (Pell, Monetta, & Paulmann, 2009a) and to achieve the valence match between affective prosody and emotional semantics of the swear word [ˈfɑ̀ːsɛn] *damn*. Acoustically matched pseudowords, [ˈvɑ́ːsɛm] and [ˈvɑ̀ːsɛm], were used to distinguish the sensory prosodic processing from the cognitive prosodic processing. In accordance with the previously established contribution of linguistic prosody to semantic processing (Zora et al., 2016, 2015), we hypothesize, firstly, that linguistic prosody will generate a larger MMN response in words than in pseudowords. Secondly, in line with previous literature (Carminati et al., 2018; Pakarinen et al., 2014), we hypothesize that a change from neutral to affective prosody will elicit an enhanced P3a response, reflecting its intrinsic motivational salience. Thirdly, we hypothesize that a unique late ERP response will be elicited by concurrent affective and linguistic prosody with the same valence, reflecting the temporal integration of affective prosody with emotional semantics of prosodic origin. According to the constructionist view, the attribution of emotions is the result of a conceptual analysis of core affect—a term used to refer to pre‐conceptual physiological states of valence, responding to motivationally salient stimuli—based on language and previous experience (Barrett, 2006; Barrett & Bliss‐Moreau, 2009; Russell, 2003, 2009; for a review see Lindquist, Wager, Kober, Bliss, & Barrett, 2012). Thus, we expect that the conceptualization of affective prosody based on linguistic prosody will be reflected in general topological principles of brain network organization (Bullmore & Sporns, 2009; Hickok & Poeppel, 2007; Rauschecker & Scott, 2009; Smith et al., 2009; Specht, 2014; Frühholz, Wiebke, & Kotz, 2016) giving rise to the unfolding of the integrative brain response. The resulting activation pattern is argued to index the continuous interaction between core affect and representations of linguistic memory (conceptual knowledge) in the production of cognitive processes (Lindquist et al., 2012).

## 2 MATERIALS & METHODS

### 2.1 Participants

Sixteen native speakers of Swedish (eight males, eight females; age range 20–47 years, *M* = 26.4, *SD* = 6.3) were recruited and tested in Stockholm. As assessed with the Edinburgh Handedness Inventory (Oldfield, 1971), all participants were right‐handed and reported normal development and hearing. All the participants gave written informed consent before the experiment, carried out according to the Declaration of Helsinki. The protocol was approved by the Stockholm Regional Ethics Committee (2015/63‐31).

### 2.2 Stimuli

The study consisted of the Swedish words [ˈfɑ́ːsɛn] *phase* and [ˈfɑ̀ːsɛn] *damn*, which are identical in segmental structure but differ in linguistic prosody, low tone, and high tone respectively, as well as in their emotional semantics, emotionally neutral and emotionally valenced, respectively. Phonologically acceptable pseudowords [ˈvɑ́ːsɛm] and [ˈvɑ̀ːsɛm], which differed from real words only in the initial and final segments, were used as controls. All the stimuli were pronounced once with an affective prosody (angry voice) and once with a neutral prosody (neutral voice). Two forms of anger have previously been reported: hot (i.e., uncontrolled) anger and cold (i.e., suppressed) anger. While hot anger tends to be pronounced with a relatively high *f*0 mean and intensity, cold anger is expressed with a moderate or low *f*0 mean and *f*0 range (Banse & Scherer, 1996; Hammerschmidt & Jürgens, 2007; Pell, Paulmann, Dara, Alasseri, & Kotz, 2009b). In the present study, cold anger was used in order to avoid the possible effects of the inherent acoustic salience of the explosive anger on the neural responses.

All the stimuli were articulated several times by a classically trained Swedish female singer and speech‐language pathologist (from Stockholm, 60 years old) in an anechoic chamber. The recordings were conducted using a Brüel & Kjær ^1^/_2_" Free‐field Microphone (Type 4189) and REAPER digital audio workstation (version 5.93), and were sampled at a rate of 44.1 kHz with 16 bits per sample. The recordings were analyzed and manipulated using an open source speech analysis software, Praat (version 6.0.33; Boersma & Weenink, 2014). In order to eliminate possible clicks, 10 ms ramps were added to both ends of the stimuli; the total duration of stimuli was 800 ms. Considering their importance in conveying the linguistic and affective functions of prosody (Banse & Scherer, 1996), intensity and *f*0 were kept constant. Pseudowords were created out of the word stimuli by a cross‐splicing technique. The initial and final segments,/f/ and/n/, in [ˈfɑ́ːsɛn] and [ˈfɑ̀ːsɛn] were extracted and replaced with their pseudoword equivalents,/v/ and/m/, which were identical with their word equivalents in manner of articulation (fricative and nasal, respectively). To preserve the natural flow of the waveform, the critical segments were extracted and spliced at zero‐crossings, and, when necessary, pulses were added/deleted incrementally to eliminate the traces of co‐articulation.

To ensure that the sound files are free from unnatural signals after manipulations and that the intended emotion was expressed successfully, a stimulus validation was performed in online research platform, FindingFive (version 1.0). Five native speakers of Swedish with a linguistics background (two males, three females; age range 35–60 years, *M* = 49, *SD* = 8.5) reported on the meaning and the emotion conveyed by prosody across all the stimuli. In contrast to previous emotion recognition studies, which used forced choice and were therefore criticized for inflating the recognition rates (Barrett, 2006; Russell, 1994), the listeners were left free in their emotion judgments and labeling. The mean accuracy for both linguistic prosody (low tone vs. high tone) and emotion (negative vs. neutral) identification was 90%.

### 2.3 Experimental paradigm

**Figure 1:**
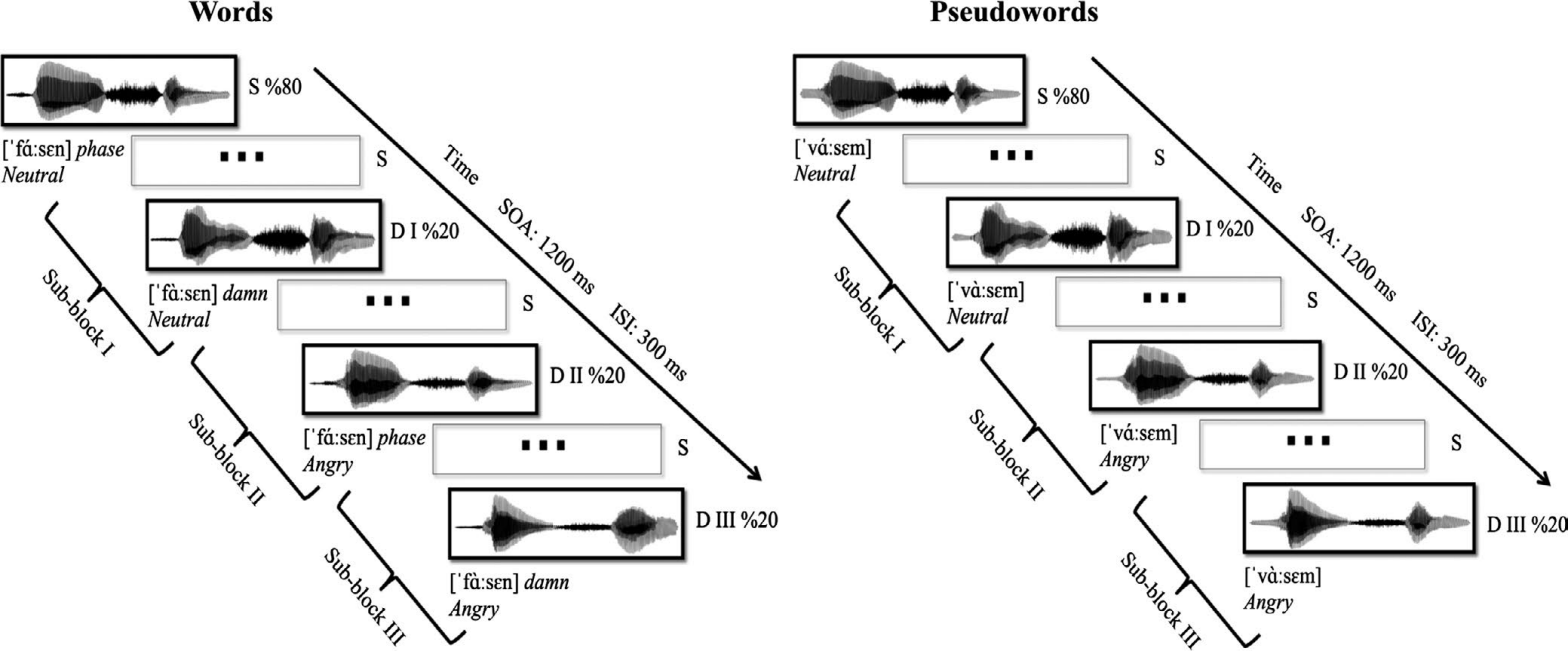
Schematic representation of auditory passive oddball paradigm. Both word and pseudoword blocks had three sub‐blocks. Stimuli that are neutral in both affect and emotional semantics served always as Standards (S). Each sub‐block represented the associated deviants and had 480 standards (*p* = 8/10) and 120 deviants (*p* = 2/10). Stimuli that are emotionally loaded but affectively neutral served as Deviant I (DI). Stimuli that are emotionally neutral but affectively valenced served as Deviant II (DII). Stimuli that are both emotionally loaded and affectively valenced as Deviant III (DIII). The stimulus onset asynchrony (SOA): 1,200 milliseconds (ms); Interstimulus interval (ISI): 300 ms

The stimuli were presented in a passive auditory oddball paradigm, where infrequent deviant stimuli (*p* = 20%) were interspersed among frequent standard stimuli (*p* = 80%). The present study had two blocks, a word block and a pseudoword block, each block consisting of 1 standard (*N* = 1,440) and 3 deviants (*N* = 360, 120 for each deviant). The paradigm is illustrated in Figure 1. The affectively neutral stimuli with low tone (i.e., *phase*, neutral in emotional‐semantic valence, pronounced with neutral prosody) and its pseudoword equivalent, always served as *Standards*; the affectively neutral stimuli with high tone (i.e., *damn*, emotionally valenced, pronounced with neutral prosody) and its pseudoword equivalent, as *Deviant I*; the affectively valenced stimuli with low tone (i.e., *phase,* neutral in emotional‐semantic valence, pronounced with angry prosody) and its pseudoword equivalent as *Deviant II*; and the affectively valenced stimuli with high tone (i.e., *damn*, emotionally valenced, pronounced with angry prosody) and its pseudoword equivalent as *Deviant III*. This design enabled us to investigate the neural responses to deviations in the linguistic prosody (Deviant I) and affective prosody (Deviant II) alone as well as in linguistic and affective prosody combined (Deviant III). The deviants within each block randomly replaced the standards, and there were at least two intervening standards between the two consecutive deviants. The stimulus onset asynchrony (SOA) was set at 1,200 ms (interstimulus interval, ISI: 300 ms).

### 2.4 Procedure

The EEG experiment was run using E‐Prime (version 2.0) in an electrically insulated and sound‐attenuated recording booth. The stimuli were delivered via loudspeakers at a comfortable listening level of 60–65 dB SPL at source. The participants were instructed to ignore the auditory stimuli, and instead watch a silent documentary (without subtitles), covering approximately a quarter of the screen. The experiment had two blocks, the word block and the pseudoword block, each having 3 sub‐blocks for each deviant (see Figure 1). The order of the blocks and the sub‐blocks was counterbalanced across participants, and the participants were given a chance to take a break between the blocks. The whole experiment, including the electrode application, lasted about 2–2.5 hr.

### 2.5 EEG recordings

The electroencephalography (EEG) data were collected with the BioSemi ActiveTwo system and ActiView acquisition software (BioSemi, Netherlands) at a sampling rate of 2,048 Hz. Recordings were made from 16 cap‐mounted active electrodes (Fp1, Fp2, F3, Fz, F4, T7, C3, Cz, C4, T8, P3, Pz, P4, O1, Oz, O2), which are equipped with pre‐amplifiers, which provide an impedance transformation directly on the electrode. The electrode positioning was in accordance with the International 10–20 system. Two additional electrodes, a common mode sense (CSM) active electrode and a driven right leg (DRL) passive electrode, were used instead of a traditional ground electrode. In addition, seven external electrodes were used: four for electrooculogram (EOG) recordings to monitor horizontal and vertical eye movements, two for mastoid recordings, and one for nose recording, which was used for offline referencing.

### 2.6 EEG data analysis

The offline EEG data analysis was performed in Matlab (version 9.4) (The Math Works Inc., Natick, Massachusetts, USA) using the EEGLAB toolbox (Delorme & Makeig, 2004). The continuous EEG data were first resampled to 256 Hz and then low‐pass filtered at 30 Hz and high‐pass filtered at 0.5 Hz. The signals were then referenced to the nose channel. To identify and remove eye artifacts, an independent component analysis (ICA), which is a computational method that separates data into statistically independent components (Jung et al., 2000), was carried out. The EEG data were then segmented into epochs from −100 to 900 ms, time‐locked to the word onset. A time window of 100 ms before the onset was used for the baseline correction. Any epochs containing EEG fluctuation exceeding ± 100 μV were automatically removed. The grand average ERPs were computed for each stimulus, and deviant‐minus‐standard subtractions were calculated for each deviant. One participant was excluded from the data analysis due to noisy data. The electrodes were grouped together in three regions of interest (ROI): Frontal, F3, Fz, and F4; Central, C3, Cz, and C4; and Parietal, P3, Pz, and P4. The time windows for ERP quantification were defined based on the grand average peaks, and the component identification was based on the previous ERP literature. Amplitudes were then computed as a mean voltage within a 50‐ms‐window centered at the peak latency in the grand average waveforms.

### 2.7 Statistical analysis

The statistical analysis was performed in SPSS (version 24; International Business Machines Corp., Armonk, New York, USA). A three‐way repeated‐measures ANOVA with factors of *ROI* (Three levels: Frontal, Central, and Parietal), *Block* (Two levels: Word and Pseudoword), and *Deviant* (Three levels: Deviant I, Linguistic prosody; Deviant II, Affective prosody; and Deviant III, Linguistic‐Affective prosody) was performed in each time window (50–100 ms, 150–200 ms, 300–350 ms, 350–400 ms, and 820–870 ms). In the case of significant interactions, follow‐up ANOVAs and *post‐hoc* pairwise comparisons with Bonferroni corrections were performed. If significant interactions occurred with the ROI, additional pairwise comparisons were carried out to investigate the lateralization effect in the relevant ROI; the electrodes were grouped in three hemispheric regions: left, mid, and right. Mean values are reported with standard deviations. Greenhouse–Geisser corrected *p*‐values are given in case of sphericity violations. Effect sizes are reported with *η^2^* (partial *η* ^2^).

## 3 RESULTS

**Figure 2:**
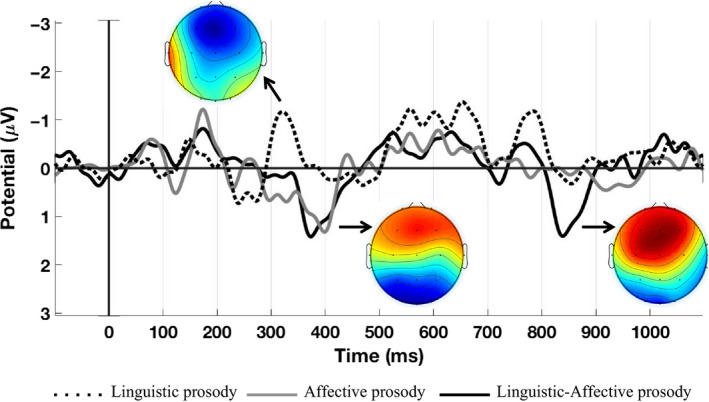
Grand average difference ERP waveforms recorded at Fz and topographic difference maps of all three deviants in word block. Dotted line, Deviant I—Linguistic prosody; Gray solid line, Deviant II—Affective prosody; Black solid line, Deviant III—Linguistic‐Affective prosody. Negativity is plotted upward. Amplitude is given in microvolts (μV) and latency in milliseconds (ms). Amplitude data entered for statistical analyses were computed from time windows that were defined based on the grand average difference waveforms [Colour figure can be viewed at wileyonlinelibrary.com]

The grand average difference waveforms (i.e., deviant‐minus‐standard subtractions) recorded from Fz and the topographic difference maps are displayed for all the three deviants (Figure 2; Deviant I, Linguistic prosody ‘neutral *damn*’; Deviant II, Affective prosody ‘angry *phase*’; Deviant III, Linguistic‐Affective prosody ‘angry *damn*’) in the word block.

**Figure 3:**
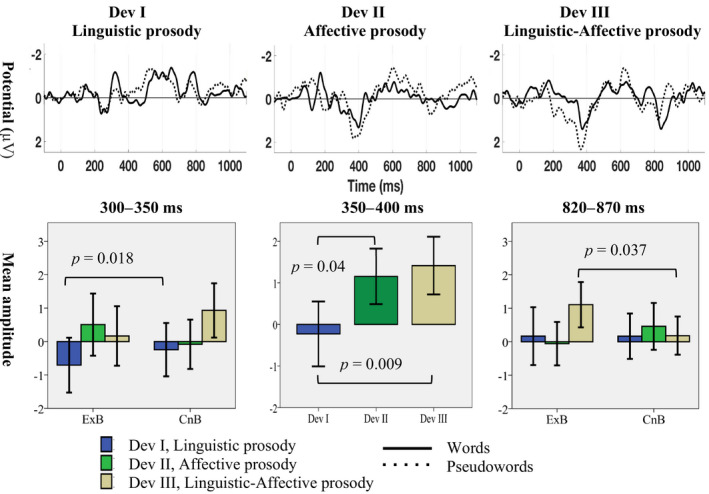
Grand average difference ERP waveforms of all three deviants recorded at Fz in both word (solid line) and pseudoword (dotted line) blocks. Mean ERP amplitudes (deviant‐minus‐standard subtractions) of Deviant I, Linguistic prosody (Blue bar), Deviant II, Affective prosody (Green bar), and Deviant III, Linguistic‐Affective prosody (Beige bar) across word and pseudowords blocks (in the left and right) as well as when these blocks merged into one block (in the middle) at three time windows. Error bars reflect 95% confidence interval. Only statistically significant *p*‐values (i.e., *p* < .05) are reported [Colour figure can be viewed at wileyonlinelibrary.com]

The results of the three‐way repeated‐measures ANOVA, follow‐up ANOVAs, and the descriptive information are presented in Tables 1, 2, 3, 4. Mean ERP amplitudes and 95% confidence intervals are displayed in Figure 3. Deviations in both linguistic and affective prosody elicited neural responses as early as about 50 ms after change onset (i.e., word onset) in both the word and pseudoword blocks. The results of ANOVA in this early latency, that is the time window 50–100 ms (Table 1), demonstrated a main effect of ROI (*F*_(2, 28)_ = 8.424, *p* = .001, *η* ^2^ = 0.376) but there were no statistically significant main effects of any other factor and no statistically significant interactions between them. The time window 150–200 ms displayed statistically significant interaction limited to the two‐way interaction of ROI and Deviant (*F*_(4, 56)_ = 3.750, *p* = .037, *η* ^2^ = 0.211), as is the case for the time window 350–400 ms (*F*_(4, 56)_ = 15.779, *p* < .001, *η* ^2^ = 0.530). Significant three‐way interaction of ROI with Block and Deviant occurs only in the time windows 300–350 ms (*F*_(4, 56)_ = 4.188, *p* = .024, *η* ^2^ = 0.230) and 820–870 ms (*F*_(4, 56)_ = 5.121, *p* = .007, *η* ^2^ = 0.268). These initial ANOVAs indicate that there are significant differences between words and pseudowords with regard to the deviants only in the time windows 300–350 and 820–870 ms (Figure 3), whereas this difference is absent in the time windows 150–200 and 350–400 ms. These time windows reveal the integrative EEG response, unfolding with the different aspects of prosody, that is, the affective, linguistic, and affective‐linguistic conditions. Further analyses of the significant interactions in these time windows are presented in the following sub‐sections.

**Table 1:** Results for three‐way repeated‐measures ANOVA with factors of region of interest (ROI, 3 levels: frontal, central, and parietal), Block (2 levels: word block and pseudoword block), and Deviant (3 levels: Deviant I, linguistic prosody; Deviant II, affective prosody; Deviant III, Linguistic‐Affective prosody) in each time window (50–100 ms, 150–200 ms, 300–350 ms, 350–400 ms, and 820–870 ms). Effect sizes are reported with *η* ^2^ (partial *η* ^2^)

Window	Factor	*F*	*p*	*η* ^2^
50–100 ms	ROI	*F*(2, 28) = 8.424	.001*	0.376
	Block	*F*(1, 14) = 0.552	.470	0.038
	Deviant	*F*(2, 28) = 2.882	.073	0.171
	ROI X Block	*F*(2, 28) = 0.706	.502	0.048
	ROI X Deviant	*F*(4, 56) = 1.783	.145	0.113
	Block X Deviant	*F*(2, 28) = 2.003	.154	0.125
	ROI X Block X Deviant	*F*(4, 56) = 0.102	.981	0.007
150–200 ms	ROI	*F*(2, 28) = 9.896	.001*	0.414
	Block	*F*(1, 14) = 0.620	.444	0.042
	Deviant	*F*(2, 28) = 0.774	.471	0.052
	ROI X Block	*F*(2, 28) = 1.136	.336	0.075
	ROI X Deviant	*F*(4, 56) = 3.750	.037*	0.211
	Block X Deviant	*F*(2, 28) = 0.217	.806	0.015
	ROI X Block X Deviant	*F*(4, 56) = 0.694	.514	0.047
300–350 ms	ROI	*F*(2, 28) = 7.008	.014*	0.334
	Block	*F*(1, 14) = 1.343	.266	0.088
	Deviant	*F*(2, 28) = 0.251	.708	0.018
	ROI X Block	*F*(2, 28) = 0.377	.583	0.026
	ROI X Deviant	*F*(4, 56) = 16.549	<.001*	0.542
	Block X Deviant	*F*(2, 28) = 1.892	.170	0.119
	ROI X Block X Deviant	*F*(4, 56) = 4.188	.024*	0.230
350–400 ms	ROI	*F*(2, 28) = 15.440	.001*	0.524
	Block	*F*(1, 14) = 3.542	.081	0.202
	Deviant	*F*(2, 28) = 2.913	.071	0.172
	ROI X Block	*F*(2, 28) = 0.091	.836	0.006
	ROI X Deviant	*F*(4, 56) = 15.779	<.001*	0.530
	Block X Deviant	*F*(2, 28) = 1.818	.193	0.115
	ROI X Block X Deviant	*F*(4, 56) = 0.361	.707	0.025
820–870 ms	ROI	*F*(2, 28) = 5.682	.028*	0.289
	Block	*F*(1, 14) = 0.020	.890	0.001
	Deviant	*F*(2, 28) = 0.866	.432	0.058
	ROI X Block	*F*(2, 28) = 1.060	.329	0.070
	ROI X Deviant	*F*(4, 56) = 5.731	.006*	0.290
	Block X Deviant	*F*(2, 28) = 2.224	.127	0.137
	ROI X Block X Deviant	*F*(4, 56) = 5.121	.007*	0.268

*p < .05.

**Table 2:** Interactions of block (2 levels: word block and pseudoword block), and Deviant (3 levels: Deviant I, linguistic prosody; Deviant II, affective prosody; Deviant III, linguistic‐affective prosody) in the time windows 300–350 and 820–870 ms in each region of interest (ROI, frontal, central, and prietal). Effect sizes are reported with *η* ^2^ (partial *η* ^2^)

Window	ROI	Factor	*F*	*p*	η^2^
300–350 ms	Frontal	Block	*F*(1, 14) = 0.992	.336	0.066
		Deviant	*F*(2, 28) = 2.713	.084	0.162
		Block X Deviant	*F*(2, 28) = 4.586	.019*	0.247
	Central	Block	*F*(1, 14) = 2.605	.129	0.157
		Deviant	*F*(2, 28) = 0.512	.546	0.035
		Block X Deviant	*F*(2, 28) = 1.647	.211	0.105
	Parietal	Block	*F*(1, 14) = 0.456	.511	0.032
		Deviant	*F*(2, 28) = 1.866	.187	0.118
		Block X Deviant	*F*(2, 28) = 0.147	.864	0.010
820–870 ms	Frontal	Block	*F*(1, 14) = 0.241	.631	0.017
		Deviant	*F*(2, 28) = 1.374	.270	0.089
		Block X Deviant	*F*(2, 28) = 5.177	.012*	0.270
	Central	Block	*F*(1, 14) = 0.089	.769	0.006
		Deviant	*F*(2, 28) = 1.297	.289	0.085
		Block X Deviant	*F*(2, 28) = 1.967	.159	0.123
	Parietal	Block	*F*(1, 14) = 0.222	.645	0.016
		Deviant	*F*(2, 28) = 1.064	.359	0.071
		Block X Deviant	*F*(2, 28) = 0.475	.627	0.033

* *p* < .05.

**Table 3:** Results for follow‐up one‐way repeated‐measures ANOVAs for two‐way interactions of region of interest (ROI, 3 levels: frontal, central, and parietal) with Deviant (3 levels: Deviant I, linguistic prosody; Deviant II, affective prosody; Deviant III, linguistic‐affective prosody) in the time windows 150–200 ms and 350–400 ms in each ROI and pairwise comparisons across deviants. Mean values (*M*) are given. Effect sizes are reported with *η* ^2^ (partial *η* ^2^)

Time window	ROI	Factor	*F*	*p*	*η* ^2^
150–200 ms	Frontal	Deviant	*F*(2, 28) = 0.127	.881	0.009
			*p*		*M*
		Linguistic prosody – Affective prosody	1.000	Linguistic prosody	–0.287
		Linguistic prosody – Linguistic‐Affective prosody	1.000	Affective prosody	–0.394
		Affective prosody – Linguistic‐Affective prosody	1.000	Linguistic‐Affective prosody	–0.393
	Central	Deviant	*F*(2, 28) = 0.652	0.529	0.044
			*p*		*M*
		Linguistic prosody – Affective prosody	1.000	Linguistic prosody	–0.371
		Linguistic prosody – Linguistic‐Affective prosody	1.000	Affective prosody	–0.520
		Affective prosody – Linguistic‐Affective prosody	0.727	Linguistic‐Affective prosody	–0.257
	Parietal	Deviant	*F*(2, 28) = 3.276	0.053	0.190
			*p*		*M*
		Linguistic prosody – Affective prosody	1.000	Linguistic prosody	–0.213
		Linguistic prosody – Linguistic‐Affective prosody	0.223	Affective prosody	–0.321
		Affective prosody – Linguistic‐Affective prosody	0.144	Linguistic‐Affective prosody	0.207
350–400 ms	Frontal	Deviant	*F*(2, 28) = 8.121	0.002*	0.367
			*P*		*M*
		Linguistic prosody – Affective prosody	0.040	Linguistic prosody	–0.227
		Linguistic prosody – Linguistic‐Affective prosody	0.009	Affective prosody	1.153
		Affective prosody – Linguistic‐Affective prosody	1.000	Linguistic‐Affective prosody	1.413
	Central	Deviant	*F*(2, 28) = 3.247	0.054	0.188
			*P*		*M*
		Linguistic prosody – Affective prosody	0.431	Linguistic prosody	–0.206
		Linguistic prosody – Linguistic‐Affective prosody	0.097	Affective prosody	0.425
		Affective prosody – Linguistic‐Affective prosody	0.990	Linguistic‐Affective prosody	0.776
	Parietal	Deviant	*F*(2, 28) = 0.241	0.787	0.017
			*p*		*M*
		Linguistic prosody – Affective prosody	1.000	Linguistic prosody	–0.442
		Linguistic prosody – Linguistic‐Affective prosody	1.000	Affective prosody	–0.627
		Affective prosody – Linguistic‐Affective prosody	1.000	Linguistic‐Affective prosody	–0.377

* *p* < .05.

**Table 4:** Results for follow‐up one‐way repeated‐measures ANOVAs for two‐way interaction of Block (2 levels: word block and pseudoword block), and Deviant (3 levels: Deviant I, linguistic prosody; Deviant II, affective prosody; Deviant III, linguistic‐affective prosody) in the frontal region of interest (ROI) in the time windows 300–350 ms and 820–870 ms. Effect sizes are reported with *η* ^2^ (partial *η* ^2^). Mean values (*M*) are reported with standard deviations (*SD*)

Time window	Deviant	Factor	*F*	*p*	*η* ^2^	Block level	*M*	*SD*
300–350 ms	Linguistic prosody	Block	*F*(1, 14) = 7.159	.018*	0.338	Word	–0.706	1.484
						Pseudoword	–0.243	1.442
	Affective prosody	Block	*F*(1, 14) = 1.891	.191	0.119	Word	0.507	1.679
						Pseudoword	–0.082	1.336
	Linguistic‐Affective prosody	Block	*F*(1, 14) = 4.063	.063	0.225	Word	0.165	1.605
						Pseudoword	0.932	1.467
820–870 ms	Linguistic prosody	Block	*F*(1, 14) = 0.000	.996	0.000	Word	0.166	1.564
						Pseudoword	0.164	1.226
	Affective prosody	Block	*F*(1, 14) = 2.130	.167	0.132	Word	–0.057	1.174
						Pseudoword	0.459	1.265
	Linguistic‐Affective prosody	Block	*F*(1, 14) = 5.331	.037*	0.276	Word	1.106	1.222
						Pseudoword	0.180	1.031

* *p* < .05.

### 3.1 Time window 150–200 ms

There is a clear negative deflection to all the deviants in the time window 150–200 ms. This negative voltage deflection is consistent with an MMN response, reflecting the acoustic deviation. The follow‐up analyses that were carried out to investigate the two‐way interaction of ROI and Deviant did not, however, yield any significant differences between the deviants in any of the ROIs (Table 3).

### 3.2 Time window 300–350 ms

There was a statistically significant difference in the mean ERP amplitude between words and pseudowords with regard to the processing of neutral (Dev I) and affectively modulated stimuli (Dev II and III), indexed by a negative deflection at around 300 ms (*F*_(4, 56)_ = 4.188, *p* = .024, *η* ^2^ = 0.230), which is limited to the frontal ROI (*F*_(2, 28)_ = 4.586, *p* = .019, *η* ^2^ = 0.247) (Figure 2; Table 2). This difference is significant only for the neutral stimuli (*F*_(1, 14)_ = 7.159, *p* = .018, *η* ^2^ = 0.338), among which words (*M* = −0.706 μV, *SD* = 1.484) elicited larger negativity than pseudowords (*M* = −0.243 μV, *SD* = 1.442; Figure 3; Table 4). A post‐hoc analysis of the lateralization of this effect did not yield any statistically significant differences in any of the hemispheric regions; left (*F*_(1, 14)_ = 0.418, *p* = .528, *η* ^2^ = 0.029), mid (*F*_(1, 14)_ = 2.349, *p* = .148, *η* ^2^ = 0.144), and right (*F*_(1, 14)_ = 1.152, *p* = .301, *η* ^2^ = 0.076). This finding is in line with an MMN response, and previous research, which indicated that the lexically relevant prosody generated a stronger MMN response in words than in pseudowords (Zora et al., 2016, 2015).

### 3.3 Time window 350–400 ms

After 350 ms, there is no statistically significant difference in the mean ERP amplitude between word and pseudoword blocks. However, both the Affective prosody and the Linguistic‐Affective prosody deviants elicit positive responses instead. This later ERP response clearly reflects the affective valence of the stimuli (i.e., Affective prosody and Linguistic‐Affective prosody; Figures 2 and 3, Dev II and Dev III, respectively), since they are clearly distinguished from the neutral stimuli (i.e., Linguistic prosody; Figures 2 and 3, Dev I). We believe that the listeners attend to the affectively valenced prosody, irrespective of the lexical‐semantic information in this time window, which explains the non‐significant difference between words and pseudowords both in two‐way (*F*_(2, 28)_ = 1.818, *p* = .193, *η* ^2^ = 0.115) and in the three‐way interactions (*F*_(4, 56)_ = 0.361, *p* = .707, *η* ^2^ = 0.025; Table 1). The follow‐up analysis to the two‐way interaction of ROI and Deviant, on the other hand, indicates that there is a significant difference between the deviants in the frontal ROI (*F*_(2, 28)_ = 8.121, *p* = .002, *η* ^2^ = 0.367). The pairwise comparisons indicate that the difference between Linguistic prosody (*M* = −0.227 μV) and Linguistic‐Affective prosody (*M* = 1.413 μV) is significant (*p* = .009) as well as the difference between Linguistic prosody and Affective prosody (*M* = 1.153 μV; *p* = .040; Figure 3; Table 3). The lateralization analysis did not show any significant results either in the left (*F*_(4, 56)_ = 3.166, *p* = .60, *η* ^2^ = 0.182), mid (*F*_(4, 56)_ = 2.571, *p* = .094, *η* ^2^ = 0.155), or the right regions (*F*_(2, 28)_ = 2.917, *p* = .071, *η* ^2^ = 0.172). The results show a significantly larger positive response to the affectively valenced stimuli (Affective prosody and Linguistic‐Affective prosody) than to the neutral stimuli (Linguistic prosody). We argue that this corresponds to a P3a response.

### 3.4 Time window 820–870 ms

The affectively valenced stimuli elicit a further clearly distinguishable positive response at 820–870 ms, when linguistic and affective prosody are combined (i.e., Dev III, Linguistic‐Affective prosody; Figures 2 and 3). In this time window, in the frontal ROI, there was indeed a significant two‐way interaction of Block with Deviant (*F*_(2, 28)_ = 5.177, *p* = .012, *η* ^2^ = 0.270). The follow‐up analyses in the frontal ROI indicated that the difference between words (*M* = 1.106 μV, *SD* = 1.222) and pseudowords (*M* = 0.180 μV, *SD* = 1.031) is significant for the deviant Linguistic‐Affective prosody (*p* = .037; Figure 3; Table 4). The hemispheric analysis effect did not reveal any significant differences in any of the three hemispheric regions; left (*F*_(1, 14)_ = 1.430, *p* = .252, *η* ^2^ = 0.093), mid (*F*_(1, 14)_ = 1.220, *p* = .288, *η* ^2^ = 0.080), and right (*F*_(1, 14)_ = 0.856, *p* = .371, *η* ^2^ = 0.058). We believe that this late positive response is a late positive component (LPC) response, which is found to be larger to pleasant or unpleasant stimuli in comparison to neutral stimuli (Cuthbert, Schupp, Bradley, Birbaumer, & Lang, 2000; Eimer & Holmes, 2007; Foti, Hajcak, & Dien, 2009; Hajcak, Moser, & Simons, 2006; for a review see Hajcak, Macnamara, & Olvet, 2010).

## 4 DISCUSSION

The main result of this study shows that when affective prosody is combined with linguistic prosody of the same valence, it generates a distinct frontal response beyond the classical MMN and P3a waves. This study provides evidence that besides distinguishing them on the basis of acoustics and semantics, the brain temporally integrates and conceptualizes these two functions of prosody based on language and experience. Moreover, this novel frontal ERP response suggests that core affect and linguistic knowledge facilitate each other in the production of cognitive processes and is in line with the constructionist view (Lindquist et al., 2012) that cognition builds on large‐scale co‐operative brain network operations (Bullmore & Sporns, 2009; Spreng, Mar, & Kim, 2009, see also Murphy, Nimmo‐Smith & Lawrence, 2003).

### 4.1 Linguistic prosody generates a MMN response

Linguistic prosody has previously been shown to generate stronger MMN response in words than in pseudowords, revealing the salience and relevance of prosodic information in semantic processing (Zora et al., 2015, 2016). Zora et al. (2016) has for instance indicated that segmentally identical Turkish words, ˈbebek ‘a place name’ versus. beˈbek ‘baby’ are distinguished on the sole basis of linguistic prosody (stress pattern), and argued that the linguistic prosody activates memory traces associated with words, and accelerates semantic processing pre‐attentively. This representation of prosody in the mental lexicon is explained by associative learning and long‐term memory representations, in line with previous studies, which indicated enhanced MMN response to words than pseudowords that do not have such representations (Alexandrov, Boricheva, Pulvermüller, & Shtyrov, 2011; Pulvermüller et al., 2001; Pulvermüller, Shtyrov, & Hauk, 2009; Shtyrov & Pulvermüller, 2002).

In the present study, a larger negative response was found to tone pattern changes at 300–350 ms. Considering the frontally maximal negativity, in line with the typical topographical distribution of MMN response (Näätänen et al., 2007), and the larger amplitude in words than pseudowords, this response is considered to be a MMN response, indexing the existence of long‐term memory traces associated with words and their prosodic features (Zora et al., 2015, 2016). Thus, when co‐activated, segments and tone patterns develop into functional long‐term memory networks that guarantee rapid semantic processing independent of attentional processes, supporting the previous literature on the representation of linguistic prosody in the lexicon.

### 4.2 Affective prosody generates a P3a response

Affectively modulated stimuli elicited a frontally maximal P3a response at 350–400 ms irrespective of lexical‐semantic information. This is in line with previous research, indicating the sensitivity of the P3a response to prosodic information, and emotional and biological arousal (Carminati et al., 2018; Foti et al., 2009; Hajcak et al., 2010; Olofsson, Nordin, Sequeira, & Polich, 2008; Pakarinen et al., 2014; Polich & Kok, 1995; Wambacq & Jerger, 2004; Wang et al., 2005), and reflecting the motivational salience of affective prosody (Wambacq & Jerger, 2004; Wang et al., 2005; see also Bradley et al., 2001; Bradley et al., 2003; Schupp et al., 2004). We speculate that the deviance from neutral to affective prosody increases the intrinsic vigilance of the participants. The difference between words and pseudowords probably disappears because the neurocognitive system is more sensitive to affect than to semantics (Pell et al., 2015), which is also in line with previous research, demonstrating a larger P3a for prosodic information compared to semantic information (Wambacq & Jerger, 2004). Irrespective of lexical‐semantic information, due to the intrinsic motivational salience of affective prosody, this ERP signature provides empirical evidence for the psychological basis of core affect (Barrett, 2006; Barrett & Bliss‐Moreau, 2009; Duncan & Barrett, 2007; Russell, 2003). Given that the constructionist model builds on basic psychological concepts (see *psychological primitives*, Lindquist et al., 2012), such as core affect, which reflects the response of visceral control systems to motivational salience, the P3a can be considered an index of motivational relevance.

### 4.3 Concurrent linguistic and affective prosody generate an LPC response

A frontally maximum positive response was elicited to the concurrent linguistic and affective prosody in the time window 820–870 ms, which is argued to be an LPC response, reflecting the valence match between emotional semantics and affective prosody. According to the constructionist approach of emotions, the attribution of emotions is the result of an automatic conceptual analysis of core affect based on stored representations of prior experiences, that is, memory and knowledge (Barrett, Lindquist, & Gendron, 2007; Lindquist et al., 2012). Accordingly, it was speculated that core affect, which was conveyed by the affective prosody and that gave rise to the P3a response at 350–400 ms, underwent a conceptual analysis later in time based on linguistic prosody and became psychologically meaningful, manifested itself as a LPC response. That is, with the help of linguistic prosody, the brain was able to conceptualize and interpret core affect conveyed by affective prosody. This is in line with a positive slow wave response, which is associated with conceptual processes such as linguistic comparison, semantic judgment and memory retrieval (Neville, Kutas, Chesney, & Schmidt, 1986; Ruchkin, Johnson, Mahaffey, & Sutton, 1988).

### 4.4 Limitations and future directions

To understand the processing of different prosodic functions, it is of essence to estimate the timing and coordination of ongoing brain activity in relation to the defined psycho‐physical stimuli, which are expected to activate different aspects of the entire brain network. The fact that a distinct LPC response arose with supra‐linear integration of two forms of prosody may have relevance for computational studies that have begun to determine the effects of functional activity on structural brain topology, suggesting that large populations of neurons across the neocortex (Ährlund‐Richter et al., 2019; Enander & Jörntell, 2019; Enander et al., 2019; Stringer et al., 2019; Wahlbom, Enander, Bengtsson, & Jörntell, 2019) work according to attractor like dynamics (Ringach, 2009). Since the constructionist approach builds on basic network operations that are neither functionally specific to language nor emotions, but common across various perceptual and cognitive domains such as executive control, memory, and language, it will be necessary to combine physiological measurements of cortical activity on different scales, that is, from single neurons and cortical microcircuits in animals, to the whole brain response by EEG and functional magnetic resonance imaging (fMRI) in humans in order to comprehend the physics of the brain's connectome. Further research is planned on comparing the effect of using passive (Opitz, Rinne, Mecklinger, Yves von Cramon, & Kruggel, 1999; Opitz, Rinne, Mecklinger, Yves von Cramon, & Schröger, 2002) and active (Buchanan et al., 2000) paradigms of fMRI, in combination with EEG, in order to better understand the interplay between and relative contribution of bottom‐up and top‐down brain network activity in the processing of prosody.

The fact that the oddball paradigm used in the present study builds on one single minimal word pair limits generalization. In order to test whether the effects truly represent a general linguistic principle (for a discussion, see Baayen, Davidson, & Bates, 2008), replication is necessary using different individual language items. Future work is therefore planned employing further homonyms with different types of prosodic semantic and affective valence, such as joy or sadness, while keeping physical and psycholinguistic parameters under tight control.

Prosody is strongly related to pitch perception (Oxenham, 2012). People with hearing loss and with cochlear implants often experience difficulties with pitch perception (McDermott, 2004; Oxenham, 2008), which affects the processing of emotional acoustic cues and their contribution to cognitive and emotional processing (Zinchenko et al., 2018). Fewer socio‐emotional acoustic cues may lead to an impoverished environment or social isolation, which in turn may contribute to delayed cognitive development or hastened decline in later life (Arlinger, 2003; Lin et al., 2013; Livingston et al., 2017; Pichora‐Fuller et al., 2016; Rönnberg, Rudner, & Lunner, 2011; Rudner, Seeto, Keidser, Johnson, & Rönnberg, 2019). Thus, it will be important, from a clinical point of view, to clarify the contributions and consequences of reduced influence of prosodic cues in both young and aging or hearing‐impaired individuals.

## CONFLICT OF INTEREST

The authors declare no competing financial interests.

## AUTHOR CONTRIBUTIONS

AKMM HZ and MR conceived the study. HZ, MR, and AKMM contributed to the design and implementation of the research. HZ performed the experiments and collected the data. HZ processed the experimental data and performed the analysis. HZ designed the figures. HZ, MR, and AKMM contributed to the analysis of the results and to the writing of the manuscript. HZ and AKMM drafted the manuscript, and MR and AKMM supervised the project. All authors contributed to and approved the manuscript. Data are available from the corresponding author on request.

## Data Availability

The data that support the findings of this study are available from the corresponding author, [HZ], upon reasonable request.
